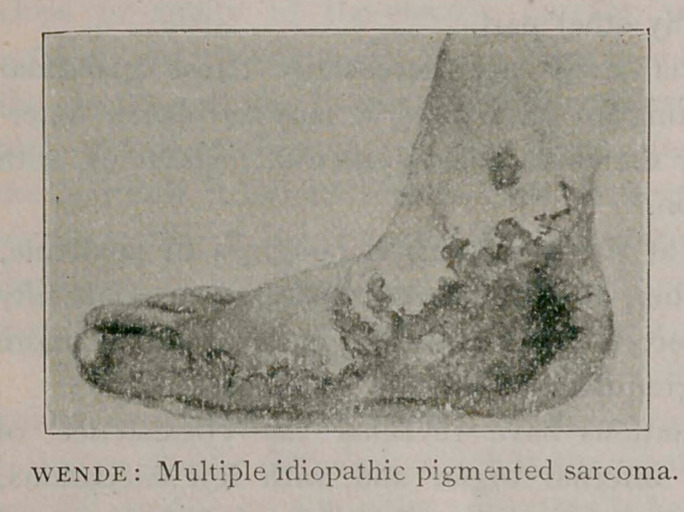# Report of a Case of Multiple Idiopathic Pigmented Sarcoma1Read at the thirtieth annual meeting of the Medical Association of Central New York, held at Buffalo, October 19, 1897.

**Published:** 1898-06

**Authors:** Grover William Wende

**Affiliations:** Buffalo, N. Y.


### REPORT OF A CASE OF MULTIPLE IDIOPATHIC PIGMENTED SARCOMA.[1]

WITH PRESENTATION OF PATIENT.

By GROVER WILLIAM WENDE, M. D., Buffalo, N. Y.

THIS peculiar and uncommon affection was first recognised by Kaposi in the year 1879 as multiple idiopathic pigmented sarcoma of the skin. His personal knowledge covers sixteen cases, all occurring in men. Since his discovery, like conditions have been

1. Read at the thirtieth annual meeting of the Medical Association of Central New York, held at Buffalo, October 19, 1897.

described by various writers. Kaposi declared that the first manifestations of the disease invariably appear on both hands and feet; then gradually progress upward, by distinct growths upon the arms and legs; and that, finally, within the course of two or three years, the trunk and face become involved. The initial growths vary in size from that of a pea to that of a large bean, and are usually colored a reddish-brown, changing in time to a bluish-red. They possess quite a firm consistence. Some are isolated and irregularly situated, some united in groups, while others coalesce into diffuse infiltrations, varying greatly in size and contour. The parts affected present a puffed appearance and the skin is often so rigid as to interfere with locomotion and manipulation. After some months the original lesions may partially or entirely disappear, leaving a pigmentation. In the process of time, other portions of the body may become affected, particularly the nose, mucous membranes, eyelids, cheeks, lips and indeed any other part.

This type of sarcoma does not necessarily cause glandular enlargement. After existing for some time, it may involve a determination toward a bloody diarrhea, hemoptysis and marasmus, with death as the consummation.

Professor De Amicis, at the international congress of medicine, held in Moscow, September, 1897, declared that there are only fifty cases of this disease on record. He also thought it would be more correct to consider it as granuloma instead of sarcomata.

Post mortem examinations have revealed the coëxistence of nodules in almost every internal organ and, with few exceptions, these new pathological formations were found to be made up of round-cell infiltrations, while the characteristic spindle-cell sarcoma was infrequent. The diffuse infiltration, peculiar hardness and the bluish-red discoloration can be readily explained by capillary hemorrhages. The prognosis is almost without exception unfavorable.

With this brief description, I shall now present to you a case of this rare and interesting malady to which my attention has been recently called. The case is not so unique in its manifestations as some, owing to the beneficial influences of treatment already received. The patient was referred to me by Dr. Bernard Cohen, who furnished the following history of the case :

Mr. —— is forty-five years of age, a native of New York City, by occupation a tailor, of spare and sinewy build and apparently well nourished. He is swarthy of countenance, with dark hair and eyes. His father died of influenza, his mother is still living at the age of seventy-five and he has three brothers and two sisters all in excellent health. As far as known, no member of the family, near or remote, was ever affected with any form of skin disease. He claims that he has never indulged in vices or excesses. His style of living has always been modest and his habits have been regular, including a close application to work. There is no evidence of syphilis. He is married and has five healthy children. His only serious ailment is the one from which he now suffers. It was in May, 1896, when he sought relief by consulting Dr. Cohen for pain in the right ankle and a feeling of distress which emanated from the stomach. The doctor attributed his condition to certain spasms due to auto-intoxication. The direct symptoms were coated tongue, large epigastrium, pain and irregular movements of the bowels. Occasionally there was some prostration, due to enervation. The urine was repeatedly examined and the color, odor, reaction and specific gravity were generally found normal; however, the sediment, consisting largely of the earthy phosphates, was increased. Neither sugar nor albumin was detected; indican frequently occurred in an unusual amount. At the first visit of the patient, Dr. Cohen observed that a section of the skin over the inner surface of the right foot presented a number of peculiar, hard, red and painful nodules which had a tendency to group. The pain has since continued. A fortnight later the doctor discovered several nodules situated in the floor of the nose which were oval and quite firm to the touch. As many as eight of these have been removed during the past year. Two months after the appearance of the original lesions, the disease manifested itself upon the index finger of the right hand in the form of circumscribed spots under the epidermis, which gradually coalesced and spread over the greater portion of the hand.

WENDE : Multiple idiopathic pigmented sarcoma.

The initial spot at one time affected the deeper structures of the skin and underlying tissue to such an extent that it resembled elephantiasis in appearance, which, upon absorption, undoubtedly due to the treatment received, gradually grew less and, at last, partially disappeared, leaving the patient lame and flat-footed. The remedy employed was arsenic, in the form of Donavan's solution.

I first saw the patient on September 2d of the present year, at which time his condition was somewhat changed. He had lost sixteen pounds in weight, his complexion was more sallow, the liver and spleen were slightly enlarged and there was constant pain in the epigastric region. The lesions were limited to the hands and feet, except a few small nodes which appeared in the nasal cavity. The sympathetic glands were not enlarged.

The manifestations found upon the right hand consisted of a dense infiltration exhibiting a dark violaceous hue, which seemed to be composed of numerous small compacted nodules located upon the dorsum and covering an area of about seven centimeters. The backs of the fingers were equally involved. Upon the palmar surface the space affected was not quite so large, measuring five and one-half centimeters. The first, second and third fingers were implicated in the distribution.

Upon the left hand, covering the upper and outer portion of the thenar eminence, existed an irregular patch five centimeters in diameter. A similar patch occupied the lower half of the little finger, which was joined to one triangular in shape and measuring six centimeters in its widest part. Upon the palmar surface and upon the anterior surface of the left forearm were four patches varying from one to one and one-half centimeters in diameter. Upon the right forearm were a few small lesions, and upon the anterior surface of the corresponding arm, three inches above the elbow-joint, was a pigmented spot having a diameter of one to one and one-half centimeters, revealing more or less infiltration.

Over the inner malleolus of the left foot a large equilateral patch of triangular shape, its sides measuring in length about twelve centimeters and its base running parallel to the plantar surface, pervaded the exterior, which was pigmented on the dorsal side, the diameter measuring three and one-half to four centimeters. A patch having an area of about two centimeters was located on the skin over the posterior extremity of the great toe, which patch contained five or six small spots of pigmentation in the border of the tarsal side. Below the external malleolus another triangular patch occupied the surface which extended backward upon the heel. Its lateral borders were seven centimeters in length and its base measured nine centimeters. Pigmentation in the spot was discernible. Two small lesions could also be seen immediately above the right knee.

The left foot, on its inner surface, was almost entirely covered with a band-like patch having a length of fourteen centimeters and whose width varied from one to four centimeters, which spread anteriorly over the toes and backward as far as the tarso-metatarsal articulations. Anteriorly over the dorsum and ankle-joint and extending upon the leg were nine distinct and separate nodes varying in size from one and one-half to two centimeters. Below the inner malleolus was recognised an area measuring from four to six centimeters.

The diagnosis of sarcoma is largely based upon the microscopical appearance of excised nodules of recent development, taken from different localities.

It was found in the present instance that the epidermal layer was apparently normal, nor did the papillæ exhibit any perceptible change in their structure, while the blood-vessels between the epidermis and subcutaneous tissue were slightly enlarged, engorged with blood and thin. Several nests of spindle-cells were easily recognised under the microscope, some of which revealed pigmented granules. The tissue between these nests was largely composed of embryonic cells. The hair follicles were unchanged.

An examination of the patient's blood, made on October 10, 1897, by Dr. Fred C. Busch, gave the following results: Hemoglobin by Fleischel, 75 per cent.; specific gravity, 1.065; number of erythrocytes per cubic millimeter, 3,900,000; number of leucocytes per cubic millimeter, 4.697; polyneuclear neuthropiles, 75.75 per cent.; large mononuclear, 12.12 per cent.; lymphocytes, 12.13 per cent.; eosinopheles, 0.

The treatment of this disease chiefly consists in the internal administration of arsenic. In the case under consideration the arsenic used was in the form of Fowler's solution combined with tincture of iron, dilute hydrochloric acid and extract of malt administered in the hope that the strength of the patient would thereby be increased and his anema more readily relieved. Since the twentieth of September the arsenic has been administered subcutaneously, according to the plan advised by Köbner. At the present time the patient is receiving daily seven drops of Fowler's solution in twelve drops of distilled water hypodermically.

Kaposi believes that the prospect of much benefit from arsenic, or any other means, is very remote. On the other hand, Kobner strongly recommends the hypodermic administration of arsenic.

Several cures due to the method advised by Kobner have been reported by other practitioners. For myself I have decided strictly to follow Kobner's plan, which is described in the *Berl. Klin. Wochen.*, No. 2, 1883, and thus far my experience shows that good results follow the employment of arsenic in the cases mentioned. It certainly diminishes tissue-changes.

471 DELAWARE AVENUE.

## Figures and Tables

**Figure f1:**